# Mild matters: trial learnings and importance of community engagement in research for early identified bilateral mild hearing loss

**DOI:** 10.3389/fped.2023.1197739

**Published:** 2023-08-08

**Authors:** Valerie Sung, Teresa Y. C. Ching, Libby Smith, Vivienne Marnane, Michelle Saetre-Turner, Alison King, Rachael Beswick, Claire E. Iseli, Peter Carew

**Affiliations:** ^1^ Prevention Innovation, Population Health, Murdoch Children’s Research Institute, Parkville, VIC, Australia; ^2^ Centre for Community Child Health, Royal Children’s Hospital, Parkville, VIC, Australia; ^3^ Department of Paediatrics, University of Melbourne, Parkville, VIC, Australia; ^4^ NextSense, NextSense Institute, Sydney, NSW, Australia; ^5^ Macquarie School of Education, Macquarie University, Sydney, NSW, Australia; ^6^ School of Health & Rehabilitation Sciences, The University of Queensland, St Lucia, QLD, Australia; ^7^ National Acoustic Laboratories, Sydney, NSW, Australia; ^8^ Hearing Australia, Doncaster, VIC, Australia; ^9^ Children’s Health Queensland Hospital and Health Service, South Brisbane, QLD, Australia; ^10^ Otolaryngology Department, Royal Children’s Hospital, Parkville, VIC, Australia; ^11^ Department of Surgery, University of Melbourne, Parkville, Victoria, Australia; ^12^ Department of Audiology and Speech Pathology, University of Melbourne, Parkville, VIC, Australia

**Keywords:** pediatric mild bilateral hearing loss, hearing amplification, newborn hearing screening, randomized controlled clinical trial (RCT), acceptability and feasibility

## Abstract

**Introduction:**

Early identification of mild hearing loss has resulted in early hearing amplification without adequate evidence of effectiveness. This paper describes learnings from a pilot trial, combined with a qualitative study, to highlight the importance of community engagement in designing research studies to determine whether early amplification benefits young children with bilateral mild hearing loss.

**Methods:**

PART 1 of the study is a proof-of-concept non-blinded multi-centre randomised controlled trial (RCT) of hearing device fitting vs. no fitting aimed to gather preliminary data and determine its acceptability/feasibility in children <2 years old with bilateral mild hearing loss.

**Results:**

PART 2 is a qualitative study to understand the barriers/enablers to RCT participation. Of 40 potentially eligible families, nine (23%) declined, three were uncontactable (7%), 26 (65%) ineligible: of these, nine (35%) did not meet hearing threshold inclusion criteria, 11 (42%) were already fitted or had made decisions on fitting hearing device, two (7%) had conductive loss and four (16%) were ineligible for other reasons. Two of 11 (18%) eligible families were randomised. With the limited sample size, outcome measures were not compared between groups. Both participants completed the trial, reported the RCT to be acceptable, and neither changed group post-enrolment.

**Discussion:**

Whilst recruitment uptake could potentially be increased by altering the eligibility criteria, better communication with and reimbursement of clinicians as recruiters, and improving awareness of the study amongst external stakeholders, the RCT methodology does not conform to family-centred practice, and potentially raises ethical concerns regarding potential adverse consequences of not offering early amplification. Parental perception of losing control over choice of management due to randomisation is not an easily modifiable factor. Alternative methodological approaches without randomisation are required to determine whether hearing amplification benefits infants with mild hearing loss.

**Clinical Trial Registration:** identifier [ACTRN12618001608257].

## 1. Introduction

Congenital hearing loss affects 1–3 in every 1,000 children, and can have adverse impacts on communication, social and emotional development, and academic outcomes in children with flow-on effects on employment and quality of life in adulthood (1). In Australia, universal newborn hearing screening (UNHS) has led to detection of any degree of hearing loss, from mild to profound, very soon after birth so that infants can receive intervention (hearing devices, cochlear implants, speech/sign intervention) early in the pre-lingual years (2). While UNHS in Australia does not aim to detect hearing loss of less than moderate degree, mild losses are being detected as a by-product. The National Workshop on Mild and Unilateral Hearing Loss defined permanent mild bilateral hearing loss as “when the diagnosis indicates there is, in both ears, a calculated or predicted average pure tone air conduction threshold at 0.5, 1, 2 kHz between 20 and 40 decibels hearing level (dB HL) or pure tone air conduction thresholds greater than 25 dB HL at two or more frequencies above 2 kHz (i.e., 3, 4, 6, 8 kHz)”(3).

Earlier detection has led to improvements in outcomes for children with moderate or greater degrees of hearing loss (2, 4–7); however, this may not be the case for children with mild hearing loss (5, 8). The studies that have examined outcomes of mild hearing loss mainly reported on school-aged children and have mixed results. Some studies reported school-aged children with mild hearing loss to have higher grade-retention rates and more dysfunction in the domains of stress, social support and self-esteem than children with normal hearing (9, 10), while other studies have not demonstrated the same (8, 11, 12). Differences in study methodology may account for some of this outcome variability. Some report outcomes only for children with slight/mild bilateral loss (11), vs. minimal (mild bilateral and unilateral combined) losses. Others recruited from populations of children known to have hearing loss (12), vs. those who reported outcomes of children with hearing loss detected via large population screenings.

A recent study of 5–7-year-olds with mild hearing loss found that full time hearing device users performed significantly better on grammar and vocabulary measures than non-users, but found no difference in articulation or speech perception (13). Other reports of school aged children, including those with mild hearing loss, have shown aided hearing can support listening comprehension (14) and oral language outcomes (15). A few studies of younger children with mild hearing loss, who received newborn hearing screening and were majority engaged with early intervention services, suggested they did as well as their normally hearing peers (16, 17). Fitzpatrick et al. assessed the outcomes at four years of a group of infants identified with unilateral and bilateral mild hearing loss; the majority (80%) of infants were recommended for amplification (17). Many parents of young children with mild hearing loss do not perceive clear benefits of early hearing amplification while others feel more positive (18, 19). There have been no randomised controlled trials (RCTs) examining the effectiveness of early hearing devices on pre-lingual children with mild hearing loss.

In the past, clinical management of mild hearing loss has relied on auditory considerations about deprivation and assessment of developmental progress in post-lingual children (20). More recently however, increasing numbers of pre-lingual infants/children with mild hearing loss are being fitted with hearing devices due to increasing detection of mild losses within weeks of birth. In 2020, Hearing Australia [the national government-subsidised hearing service provider for all children and youth aged 26 years and under in Australia (21)] recorded that 56.7% of hearing device fittings in Australian children less than 2 years old had mild hearing loss in the better ear of ≤40 dB HL (22). Two recent studies examined the parental and audiologist perceptions of early management of mild bilateral hearing loss. Parents reported significant stress around the diagnostic processes, guilt about the potential future negative effects of not fitting hearing devices for their infants, and a multitude of challenges around hearing device compliance and maintenance (18). Many parents felt the decision for hearing device fitting was often left up to them to make (18). This was reflected in audiologists reporting that they considered multiple child and family-related factors and the perspectives of parents and families in making decisions about fitting in this population (23). Indeed, audiologists perceived the clinical management of these children to be challenging, mainly due to the lack of evidence to guide management (23).

Evidence on the effectiveness of early hearing device fitting in infants and pre-lingual children with mild hearing loss is therefore needed to guide management of these children, especially in the face of healthcare costs and potentially significant burdens for these families and society. We attempted to answer this research question through a proof-of-concept RCT aimed to gather preliminary data, to be used towards planning for a possible future more definitive RCT, to compare, in children less than 2 years old with bilateral mild hearing loss, language outcomes of those fitted with hearing devices vs. those without hearing device fitting, 6 months post-randomisation. The secondary aims were to collect 6 months post-randomisation data on child social abilities, functional performance and listening effort, parental morale, parent-child relationship and quality of life, as well as determine the acceptability and feasibility of the RCT. However, as our trial failed to recruit sufficient participants, we engaged clinical audiologists and families of young children with mild bilateral hearing loss to conduct a qualitative study to understand the barriers/enablers to RCT participation (PART 2). This paper overall aims to describe our learnings from both the RCT and the qualitative study to highlight the importance of community engagement to help develop the impetus, design and implementation of future research studies to determine whether early amplification benefit young children with bilateral mild hearing loss.

## 2. Materials and methods

### 2.1. PART 1: randomised controlled trial

Here, we describe the essential details of our trial's recruitment methodology in the context of an unsuccessful trial from which key lessons were learnt. The RCT is registered with the Australian and New Zealand Clinical Trials Registry (ACTRN12618001608257); the full protocol is available on the ANZCTR website. The study has ethics approval from the Royal Children's Hospital Human Research and Ethics Committee HREC 38112 (HREC/45275/RCHM-2018-151266).

We set out to conduct a proof-of-concept non-blinded multi-centre RCT comparing hearing devices (intervention) with no hearing devices (control) in children less than 2 years old with bilateral mild hearing loss (21 to 40 dB HL) across at least 3 octave frequencies between 250 and 4000 Hz by objective or behavioural testing. Recruitment occurred in three states in Australia: Victoria (VIC), New South Wales (NSW) and Queensland (QLD). Children must have met all of the following criteria to be enrolled in the study: (a) born in VIC, NSW, or QLD and eligible for services of Hearing Australia (Australian resident/citizenship status), (b) less than 2 years old, (c) had parents/carers who spoke English adequately to give consent, (d) had, within the last 3 months, been confirmed to have bilateral mild hearing loss (21 to 40 dB HL) across at least 3 octave frequencies between 250 and 4,000 Hz by objective or behavioural testing, and (d) had pure sensorineural hearing loss. Children with any of the following criteria were excluded from the study: (a) families who had already made a decision of fitting/not fitting hearing devices for their children, or children who were already fitted with hearing devices, (b) complex medical problems/major disabilities (e.g., recurrent seizures, major cardiac problems requiring multiple operations), (c) any conductive hearing loss, (d) hearing threshold of <21 dB or >40 dB HL at any frequency, (e) medical contraindication to hearing device fitting, and (f) families who definitively planned to move, during the following 6 months, to a location where follow-up assessment was not possible or practical.

Six months after the study started, in response to the poor recruitment rate, inclusion criteria for hearing thresholds were broadened to the following: had, within the last 3 months, been confirmed to have bilateral mild hearing loss [at least three frequency average ≤40 dB eHL (estimated hearing level) between 250 and 4,000 Hz] by objective or behavioural testing. That is, children with three- or four-frequency average threshold of <21 dB or >40 dB HL were excluded.

The study was conducted at Hearing Australia clinics in the three states from 1st February 2019 to 31st January 2020. At enrolment, the participant child was randomised to either intervention or control. Children in the intervention group received hearing devices as per standard Hearing Australia protocol, and were followed up for hearing device compliance (monthly parent-report via a five-question survey sent to the parent's mobile phone or email, and hearing device data logging) over 6 months. They also received normal audiological care and parental support as clinically required following Hearing Australia protocol over 6 months, including assessments and clinical counselling by a Hearing Australia audiologist regarding hearing devices fitting, fitting adjustment and follow up appointments at the Hearing Australia centre (21). Timing of fitting, model of hearing devices and clinical care were determined by the Hearing Australia audiologist. Children in the control group were not fitted with hearing devices and received normal audiological care and parental support as clinically required following Hearing Australia protocol over 6 months, including assessments and clinical counselling by a Hearing Australia audiologist.

The randomization methodology, primary and secondary outcome measures are available from the ANZCTR website and are not reported here as they are not the focus of our learnings in this paper. The feasibility of the RCT was measured by the: (a) number of children enrolled as a proportion of eligible children; (b) number of children who dropped out as a proportion of enrolled children; (c) number of children who changed treatment group from original treatment allocation, as a proportion of enrolled children; and (d) device use—measured by automated data logging in hearing devices over 3 months, and monthly parent report on proportion of device use during waking hours over the last week, during the 6 months after fitting. The acceptability of the RCT was measured by parent-report at the 6 month follow-up through survey with the following questions: (a) “How do you feel about your child being allocated to the hearing aids group vs. the no hearing aids group?”, (b) “Overall, do you feel your child has been advantaged or disadvantaged by being assigned to a fitting or no fitting group?”, and by free text responses.

We had estimated our expected sample size according to the known incidence of mild bilateral hearing loss [0.4/1,000 newborns (24)]; approximately 100 infants would be eligible from all 3 states over one year. Anticipating a consent rate of 60% and a drop-out rate of 20%, approximately 48 children with mild bilateral hearing loss would be enrolled in the RCT over the study period of one year, with approximately 24 in each intervention arm. The expected recruitment numbers per site were ∼15 from VIC, ∼18 from NSW and ∼15 from QLD. Data collection was via REDCap.

### 2.2. PART 2: qualitative study

Subsequent to determining the feasibility of the RCT, a qualitative study was undertaken to explore the factors that influenced parental uptake of the RCT. This qualitative study received ethics approval (as above). Over a four-month period from June 2020 to September 2020, we invited caregivers who met the same eligibility criteria for the RCT, but without excluding those who had already made a decision of fitting/not fitting hearing devices for their child, to participate in a semi-structured phone interview and complete a basic demographic child and parent questionnaire via REDCap (see Supplementary Material Interview Guide). Due to COVID-19 restrictions in place at the time of data collection, face-to-face interviews could not be offered. During the 4-month period, we also invited by email diagnostic and rehabilitation audiologists to participate in semi-structured phone or videoconference interviews to explore their perceptions of factors that influenced parental uptake of an RCT. Audiologists also filled in a brief demographic questionnaire via REDCap about their audiological experience and frequency of managing children with mild hearing loss. For both caregiver and audiologist groups, data collection continued until no new themes emerged (saturation of themes). This was verified during data collection through reflective discussion after each interview between the researcher conducting the interview and project team members. Two researchers conducted the interviews, one completing the caregiver group and the other completing the audiologist group. Interviews were transcribed using a third party transcription service, and transcriptions were reviewed by the researchers who conducted each interview for accuracy and to allow reflexive thought to identify any assumptions the researcher may bring to the research purpose (25).

We theorised that factors influencing parental uptake in a RCT worked together to influence the decision-making process, therefore Grounded Theory methodology was applied for analysis of all interviews together. Grounded Theory is an iterative, inductive methodology that results in the generation of a theoretical explanatory process relating to a phenomenon, in this case the parental decision making process (26). In a similar approach to other qualitative analysis methods such as thematic analysis, interview transcripts were coded to categorise and assign meaning to data to allow the identification of similarities, differences, and patterns. Through iterative processes the coding was organised within a framework that denoted interactions between concepts (27).

The same researchers who completed the interviews independently completed initial coding of each interview transcript, and the two researchers discussed areas of discrepancy until consensus was reached on how to complete initial coding of all transcripts. A further six transcripts underwent initial coding and discussion held with the wider research team (VS, TC, VM, LM, MS, RB) to ensure consensus on initial codes before all transcripts were coded. After initial coding was completed, a small group of researchers met (VM, MS, LM) to undergo intermediate coding. Intermediate coding is the process of identifying categories and concepts from the initial codes and beginning to identify relationships between the concepts for a theory to merge from the data. The initial theory on parental decision making to participate in a RCT was discussed with a wider research team (VS, TC, LS, VM, LM, MS, RB) and then refined in the final stage of advanced coding, where the final framework was derived and interrelated concepts were established.

## 3. Results

### 3.1. PART 1: RCT

**Figure 1:**
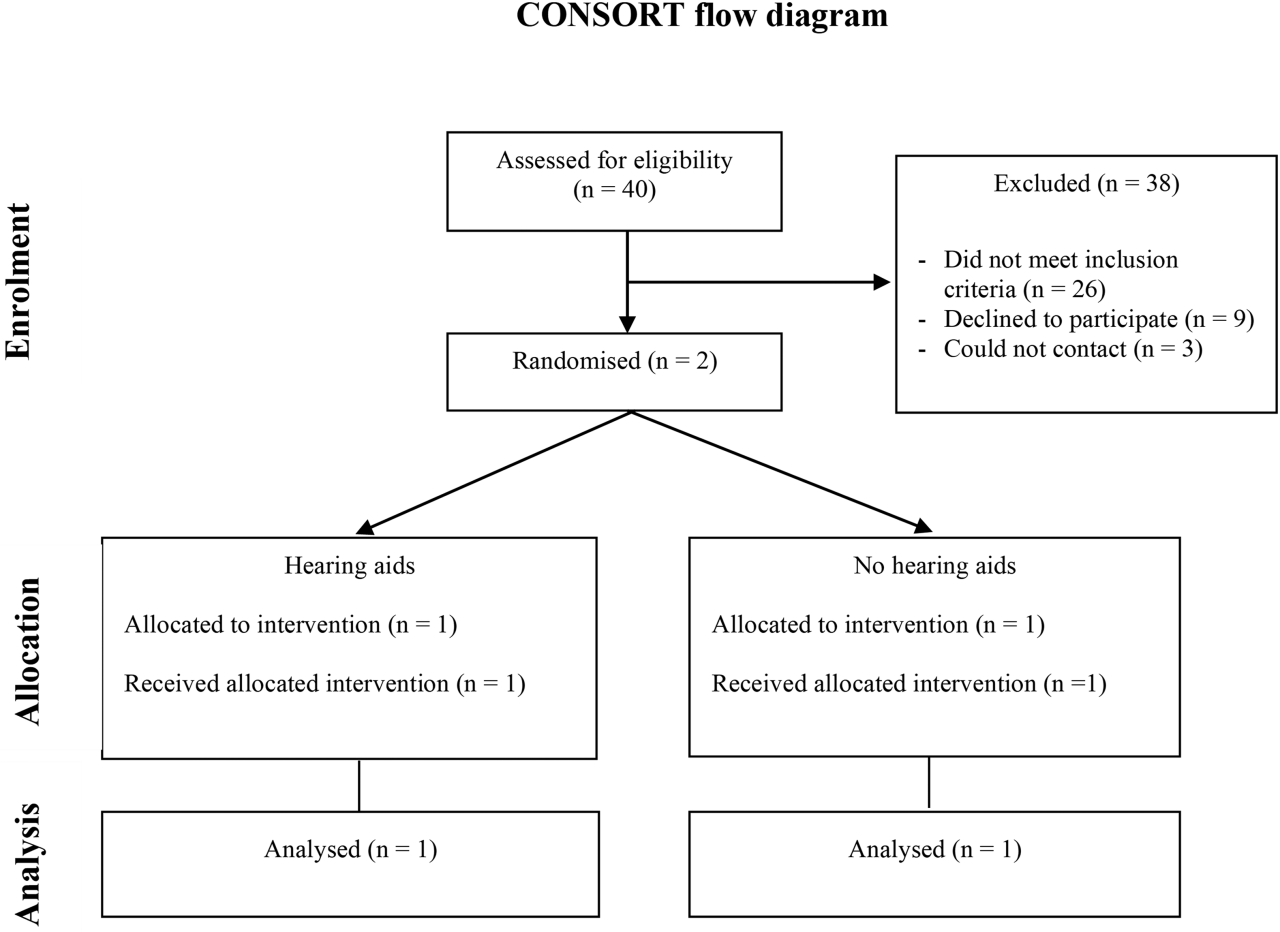
Participant flowchart.

Here we report only on the outcomes of recruitment for the RCT in view of the aims of this paper. Forty infants were referred to the study. Fifteen referrals were from VIC, 16 from NSW, and 9 from Queensland. All referrals were from audiologists or the Victorian Infant Hearing Screening Program. No referrals were from ENT specialists or paediatricians. Of the 40 potentially eligible families, 3 could not be contacted, 26 were ineligible and 11 were eligible. Of those eligible, 2 participated and 9 declined (Figure 1).

Table 1 shows the reasons for ineligibility. The main reason for ineligibility was not meeting the audiometric threshold criteria (35%). The study team identified this as an issue halfway through the recruitment period and relaxed the inclusion criteria. Six families (23%) had already made the decision to fit hearing aids or had decided that they did not want hearing aids. Five infants were already fitted with hearing aids (19%). Other infants were excluded because the family had insufficient English to consent, were moving away, or the infant had conductive hearing loss, or had complex medical issues contraindicating hearing aid fitting.

**Table 1:** Reasons for ineligibility (total *n* = 26).

Reason ineligible	Frequency (%)
Did not meet hearing threshold inclusion criteria	9 (35%)
Already decided to fit/not fit hearing aids	6 (23%)
Already fitted	5 (19%)
Conductive or other hearing loss	2 (7%)
Insufficient English	1 (4%)
Complex medical	1 (4%)
Moving away	1 (4%)
More than 3 months since diagnosis	1 (4%)

Two infants participated. One was randomised to the intervention “hearing devices” group and the other to the control “no hearing devices” group. Both participants were followed up at 6 months. Follow-up of outcomes concluded by 16th April 2020. The participant characteristics, and the primary and secondary outcomes for each participant, are reported in the Supplementary Tables. We did not compare primary and secondary outcomes between the two groups due to the limited sample size.

The following describes the outcomes of the feasibility measures:

• 2 out of 11 (18%) eligible participants consented to take part.
• Neither of the two participants dropped out of the study.
• Neither of the participants changed treatment group from the original treatment allocation.
• Device use: The participant parent from the intervention group completed 5 out of 6 monthly surveys on device use. The child was ill during two of the reporting weeks and the parent indicated they were not typical weeks for device use, so these two weeks were excluded from analysis. By parent report, devices were worn on average by the participant for 47% of waking hours on weekdays (3.5 h), and 21% of waking hours on weekend days (1.7 h). This is compared to data logging indicating the participant wore the devices on average 4.6 h (right) and 4.5 h (left) per day over 3 months of the study period.

Both participant families filled in surveys about the acceptability of the study. They reported positive or neutral feelings about participating in the study. The intervention family reported feeling positive about their child being allocated to that group and felt their child was “highly advantaged”, commenting that being assigned to the fitting group was “good for his learning development”. The control family felt “neutral” about being allocated to that group and did not feel advantaged or disadvantaged. They commented that they were glad their child “didn’t have the trouble of a hearing aid” but were also “worried that it might have been good for her”. They also indicated they understood the purpose of the study well with the comment “I suppose that's the whole point of the study—we just don't know what's best!”.

### 3.2. PART 2: qualitative study

#### 3.2.1. Recruitment

Ten caregivers and 11 audiologists completed interviews. Tables 2, 3 outline the demographic characteristics of both groups.

**Table 2:** Demographic characteristics of child and caregiver for interviews.

ID	Gender	Age of child (months)	Fitted with hearing aids	Parent with hearing loss	Primary language spoken at home	Caregiver completing interview
P201	Male	2	Yes	Father	Cantonese	Father
P203	Female	9	No	Mother	English	Mother
P205	Male	3	No	Father	English	Mother
P206	Male	3	No	No	Maltese	Mother
P306	Female	3	Yes	No	English	Mother
P311	Female	2	No	No	Greek	Mother
P312	Female	1	No	No	English	Mother
P401	Male	3	No	No	English	Mother
P402	Female	4	No	No	English	Mother
P404	Male	4	Yes	No	English	Mother
P405	Male	5	Yes	No	English	Mother

**Table 3:** Demographic characteristics of audiologist completing interviews.

ID	Gender	Age Group (years)	Area of practice	State	Number of years peadiatric audiology experience	Number of children with bilateral mild hearing loss seen in last 12 months
A201	Female	31–40	Rehabilitation	NSW	5–9	≤5
A202	Female	41–50	Rehabilitation	NSW	15–19	≥15
A300	Female	≤30	Mixed	VIC	15–19	6–10
A307	Female	31–40	Rehabilitation	VIC	5–9	≤5
A309	Female	51–60	Diagnostic	VIC	20+	≤5
A310	Female	51–60	Diagnostic	VIC	20+	≤5
A312	Female	31–40	Rehabilitation	VIC	15–19	≤5
A400	Male	≤30	Rehabilitation	QLD	5–9	6–10
A403	Female	41–50	Rehabilitation	VIC	2±	11–15
A405	Female	≤30	Rehabilitation	VIC	<5	≤5

#### 3.2.2. Interview outcomes

**Figure 2:**
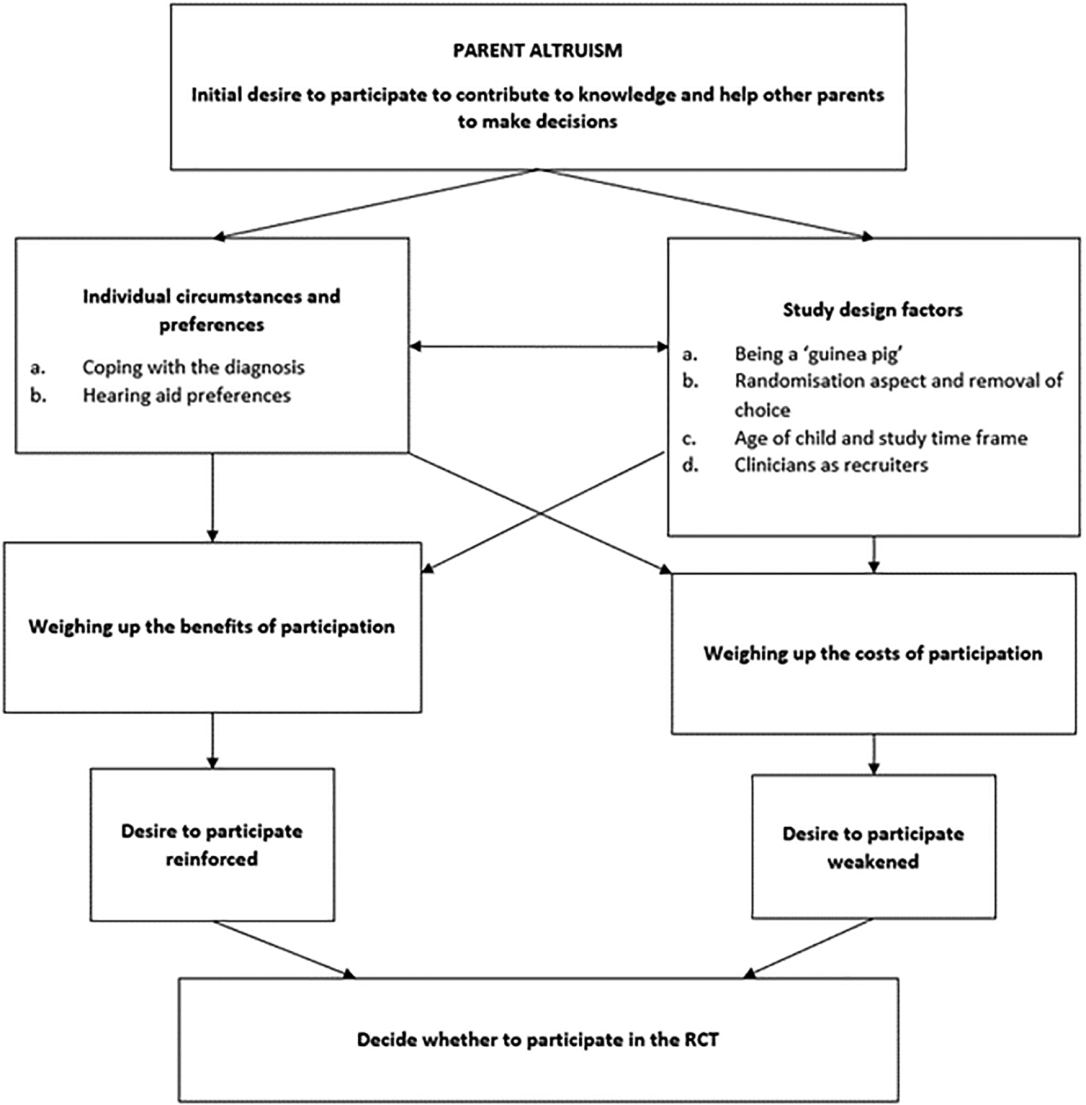
Overview of decision making (figure adapted from McCann et al. (2010) (28)).

The decision that a parent would ultimately make was the result of a complex interplay of: (1) individual circumstances and beliefs, (2) study design factors, (3) perceived benefits of participation, and (4) perceived costs of participation. These four themes and their corresponding subthemes generated a decision-making framework that was underpinned by a major theme of “parent altruism” that was common across interviews and represented in Figure 2.

##### 3.2.2.1. Overarching theme: parent altruism

Overwhelmingly, parents spoke of the challenges they faced when making decisions about hearing amplification for their child. Perhaps unlike parents of children with more severe hearing loss, parents of children with mild hearing loss experienced “overwhelming indecision around whether to get hearing aids” [P401]. Parents felt the need to do the right thing by their child:

*Family say, “Well just do what*”*s best for her”, and I was like, yeah, well I'm gunna, but I didn't know what that is* [P312].

Many parents had experienced high levels of stress making decisions around hearing aid fitting due to the lack of evidence surrounding the effectiveness of fitting hearing aids for this population. As such, a common theme was feeling inclined to participate in the trial for altruistic reasons, or “the general good of everyone” [P201]:

*When we were trying to make the decision about whether to get aids or not, basically it felt really hard to make the decision because there was no information and there was no studies that were really conclusive. I'd want to participate so that future people could make the decision easier.* [P401]

Similarly, audiologists with experience attempting to recruit parents into the trial described how the uncertainty surrounding whether to fit hearing aids would lead parents to act altruistically. Parents would “*jump on anything they can do to help”* [A405]:

*I found that sometimes they're a little bit easier to recruit only because there's that ‘Do we fit? Do we not fit?’ Some families are like ‘Oh, I’d really love to know. I'd love to be part of this, if it makes that decision easier for somebody else down the track.'* [A201]

The desire to help future parents was often the first factor mentioned when discussing trial participation. When probed further on their decision-making process, parents would relate their reasoning to this anchoring argument, either reinforcing or weakening the initial desire to participate. This is illustrated in the decision-making overview in Figure 2 and explained further through the remaining themes and subthemes.

##### 3.2.2.2. Individual circumstances and preferences

During the interviews, parents and audiologists described how a family's circumstances and preferences would influence their willingness or capacity to participate in the trial. Two subthemes were identified: timing of study presentation, and hearing aid preferences.

###### 3.2.2.2.1. Study timing: during coping with the diagnosis

Recruitment into the hypothetical trial and subsequent randomisation (fitted or not fitted with hearing aids) would have occurred when the child was very young, soon after the diagnosis of hearing loss was made. Consideration of the context surrounding families and their emotional state at this point in time demonstrates how the time at which recruitment is conducted can affect uptake. Parents described how receiving their child's diagnosis was an overwhelming experience. In the early stages post-diagnosis, parents felt bombarded by information, needed to attend multiple appointments, and some struggled to come to terms with the diagnosis:

*As soon as the diagnostic came through, I received numerous emails with attachments that I can read through the hearing loss. But then like those things to me are—I know it's factual but, still, it doesn't really feel very real to me.* [P201]

Some parents found the invitation to participate in a study brought the emotions surrounding diagnosis and the decision about providing amplification to the forefront. The timing of study presentation is a factor that likely influenced uptake of the trial:

*So, I guess there's so many decisions, like so many different appointments and things we go through just to get to this stage and you just had a baby. There's just so much going on. It [participating in research] feels like an extra thing without even starting to think about making that decision, and I guess like we're right now in the decision process of whether to get hearing aids or not and we're trying to get as much information as we can to make that decision. I feel like we're half agonising over the decision… Maybe in like another month I would maybe think about it [participating].* [P401]

###### 3.2.2.2.2. Preferences for fitting or not fitting

Another factor that influenced parent willingness to partake in the trial was attitudes towards fitting of hearing aids. Preferences for fitting or not fitting varied among the interviewees; some had strong preferences either way, and some had no preference or had not yet made up their mind. One parent described how participating in the trial would make her feel anxious about her child's development:

*I think my major thought process would be I'm someone who's very for hearing aids. So, my thought process would be—if he was chosen not to have hearing aids, would that impact his language or development in the future and things like that? I've always put it as if there was a gap or a little stone missing in your steppingstones, if I can replace that for my son I will. So, yeah, it would probably be quite anxious for me. I don't think I would actually join something like that, just in case we were picked to not have them, because I wouldn't want any negative effects on his development or hearing or language.* [P405]

Somewhat unexpected was that even when parents had strong preferences for fitting or not fitting, this did not necessarily mean they would decline participation in the trial. One parent (who had decided against hearing aids for her child) described how her personal experience of hearing loss had influenced her preferences for her daughter:

*Well for me being hearing impaired, like it's a little bit confronting to see your daughter with hearing aids and knowing that the stigma around wearing hearing aids at times with other kids and other people that don't really understand.* [P203]

However, this parent suggested that she would not decline to participate and would not be disappointed being randomly selected for the fitted group as she understood “*that it's for the purpose of the study”* [P203]. This highlights the importance of ensuring that parents have a good understanding of the purpose and benefits of a study and its design.

##### 3.2.2.3. Study design factors

This theme speaks to the challenges faced by researchers when attempting to strike a balance between ensuring that a study design can answer a research question using the highest possible level of evidence, whilst simultaneously maximising participation rates. The responses from parents and audiologists demonstrate that researchers should have clear reasons for the choice of study design, and this reasoning should be made available to recruiters as well as to participants. The researcher conducting the interviews noted that for parents without a background in research or experience participating in trials, the nature of a randomised control trial was understandably foreign. This theme has four subthemes, (1) being a “guinea pig”, (2) randomisation aspect and removal of choice, (3) age of child and study time frame, and (4) clinicians as recruiters.

###### 3.2.2.3.1. Being a “guinea pig”

A common concern voiced by parents was that their child would be participating in something where the outcome or long-term effect was unknown:

*I do think people usually have a tendency of being scared of trying to do things like this. Like, they feel a bit like they're guinea pigs, I would say. Like, why should I partake in an experimental study, or why should I kind of put my information, all that, out there?* [P311]

*Thinking about it from a child's perspective, realistically they're like a guinea pig, using them—will this work, will that not work, how is this going to affect them, will it not affect them at all?* [P405]

Parents stated that the complex nature of the study design and the uncertainty could have an impact on parental uptake:

*Clearly a lack of knowing… the lack of knowledge, like, of knowing what the study is and what the whole process is, would probably make other people around parents suggest not to, not to do it.* [P311]

When parents had a good understanding of the importance of the research, and indeed the benefits that a randomised control trial design can give, they were more supportive:

*My husband and I, we're both scientists and we kind of know that research and studies like this are needed to find out things that are going to help people and the greater good.* [P203]

###### 3.2.2.3.2. Randomisation aspect and removal of choice

Perhaps the most pertinent study design factor that parents and audiologists commented on was the randomisation into the fitted and non-fitted group. Hearing aid preferences varied across interviewees, and it seemed as though some parents were not comfortable having the decision regarding devices taken away. In addition, it raises a potential ethical concern with not providing amplification for these children when failure to do so could potentially negatively impact communication development:

*You're basically signing up to get the decision taken off you. It feels a bit scary. Like you might be doing the wrong thing by your kid if you got allocated not and your child might be six months behind if they don't have them, but they actually needed them… I guess if I had to commit to a decision for six months, I wouldn't want it to be random, so I probably wouldn't take part in it* [P401].

Audiologists also noted that some parents were not comfortable with randomisation:

*When you're doing these random assignments I know that some parents don’t like that because it's taking the control away from them* [A202].

One audiologist suggested that for parents who were undecided or struggling with the decision surrounding hearing aids, the removal of choice can be positive:

*I found it especially helpful offering a research project or trial like that, offering it to the families that were very undecided, those that were really in two minds. They had absolutely no idea which way they were going to go. This [participating in trial] meant that they didn't have to decide. It was kind of decided for them* [A400].

###### 3.2.2.3.3. Age of child and study time frame

Another factor influencing participation in an RCT was the relatively short time frame of the study, and the age requirement. Parents generally felt comforted that their participation would be “*only six months”* and they could go back to their original decision about hearing aids or change their mind after this period [P203]. Similarly, a number of parents thought the impact of participation would be minimal due to their child's young age:

*Because I still think at his early months—he's only not even three months—to have without the hearing aids for a period of up to six months, it probably won't hurt him that much as compared to when he is two years old, three years old or at school age.* [P201]

One parent suggested six months was a long period of time, and that the commitment could “become a bit of a burden” (401). However, the opportunity for closer monitoring of the child's development was a strong benefit to participation for this parent, and it would factor in to whether she would agree to participate:

*I guess if you're involved your child's got more touch points and getting more monitoring that could help identify if there was an issue because I'm assuming if it was identified that he really was not hitting milestones, it'd be easier… I guess people checking in on him and seeing how he's progressing.* [P401]

As will be discussed in the following theme, the support of audiologists for the study design would be important in this context since clinicians were recruiting families. Some audiologists voiced their concern over the study design:

*It's good in that it's not a huge length of time to commit to. I don't know how much difference six months would show given they are quite young and they are still so close to the parents and all that kind of stuff. So I don't actually know if it would be long enough to show any significant differences or not* [A201].

###### 3.2.2.3.4. Clinicians as recruiters

Due to the relationship with the hearing service provider, audiologists working clinically were tasked with recruiting participants into the study. This could be more effective or beneficial than researchers as researchers are unfamiliar to families, and would not attain as high level of trust as clinicians for facilitating the informed consent process. However, having someone outside the research team responsible for recruitment adds further complexity to the factors that can influence parental uptake of a trial. Like parents, audiologists were generally supportive of the research because they had experienced the challenges of providing recommendations without definitive evidence:

*I think it would give clinicians more confidence in knowing which way to advise parents about the benefits of amplification* vs. *non-amplification, or any kind of intervention, I guess because right now it's really hard. I find it very difficult to sort of know what to say to parents* [A300].

Nevertheless, audiologists have a relatively short time with families and were justifiably focused on their main responsibility of providing family-centred clinical care. As one audiologist put it: “*there's a lot to fit into that appointment time”* [A403]. Some would simply forget to recruit, “*because in the middle of a diagnosis that's the last thing on your mind”* [A300], and others spoke of recruitment potentially undermining the recommendations they were making:

*So if I'm having difficulty convincing them to go through with various recommendations, I would probably not add ‘And would you like to participate in a study?’ into the mix either, and because I think it undermines the recommendation. It's like ‘I’m recommending that you go and get some hearing aids, and by the way, we don't really know yet whether it's going to make a difference.' It's like, yeah, maybe not.* [A310].

Audiologists had the best interests of the family in mind, and wanted to make sure that participating in the trial would benefit the family:

*I think either way just being involved with the study, I'd be happy with that because I know that even the children that were randomly allocated into the unaided group, they still received ongoing reviews and speech assessment at certain periods. So, they weren't necessarily just left with nothing.* [A400]

It was clear that utilising clinicians as recruiters meant that audiologists had to balance their clinical role with their recruitment responsibility. Clinicians have the benefit of knowing how the family is coping with the diagnosis and other priorities in their life, and some stated they would select which families they would attempt to recruit based on how they were “managing the news and the diagnosis” [A310]. Most, however, were of the view that they generally “wouldn’t deny anyone the knowledge of the research” [A309]. Unlike a member of the research team, clinicians may not always prioritise recruitment, particularly if they had less understanding of, or experience with, research:

*I think that because I have worked in research before I have an appreciation of the benefits of research as well as the challenges of recruitment. So I feel like most other audiologists wouldn't probably give as much energy to this sort of thing as I would. I would probably be more pro supporting research than the general audiologist, and I would find the time, but I don't think that a lot of other audiologists would* [A403].

## Discussion

Our paper highlights the importance of community engagement in designing and conducting research to determine whether early amplification benefits infants and young children with bilateral mild hearing loss. Important lessons have been learnt from the failure of recruitment for our proof-of-concept RCT. Our subsequent qualitative study explored the barriers and enablers of participation in a RCT, and identified useful concepts that could be applied to future research studies that attempt to address the research question.

Over one year from 1st February 2019 to 31st January 2020, 40 infants were referred to the RCT, which was much fewer than expected. During this period, according to data from hearing screening programs and diagnostic audiology services, approximately 146 children were diagnosed with bilateral mild hearing loss in the three states; therefore, only approximately 27% (40/146) were referred to the study team. There were a few possible reasons for why 106 infants were not referred to the study. First, and anecdotally the most common reason, diagnostic and rehabilitation audiologists indicated that they did not refer families to the study if they did not meet the audiology threshold criteria (e.g., infants originally diagnosed with mild bilateral hearing loss may subsequently have normal hearing or moderate hearing loss). Second, a database error in Victoria accounted for 9 potentially eligible families missed from being referred. Third, audiologists indicated that they did not refer families if families had insufficient English to give consent.

Of the potentially eligible participants referred, the majority were ineligible (26/40, 65%). The main reason was infants not meeting the hearing threshold criteria. Our inclusion criteria depended upon the infant meeting particular thresholds of hearing loss over three or four frequencies in two ears. Infants may be diagnosed with bilateral mild loss, but even if a single hearing threshold in one (or both) ears changed on subsequent testing, they may have been assigned a different degree of loss. These infants would then have become ineligible for the study. This reflects the fact that diagnosis of mild hearing loss can be uncertain and challenging, and often requires multiple diagnostic audiology appointments to confirm the hearing status (16). Conversely, infants initially diagnosed with a different degree of loss may subsequently become eligible, but we may have missed the window to recruit them.

The other main reason for ineligibility was the infant having already been fitted with hearing aids (4/26, 19%), or the family having already made the decision to fit or not fit hearing aids (6/26, 23%), at the time when they were approached by the researcher. Nine out of 11 eligible families declined to take part (9/11, 82%). Parents did not want their child's treatment decision to be randomised, or did not want to be involved in research. The high ratio of families who declined compared to those who participated indicates that there may have been considerable barriers to participation, and could possibly reflect parents' preference to make their own choice in hearing device fitting. There are many possible reasons for this: parents may feel empowered to take action for their child's hearing loss by fitting hearing aids; parents may perceive benefits to hearing device fitting; and parents may feel potential guilt of denying the child the opportunity to access a full range of sounds, especially if the child has subsequent language delays (16). These factors were evident from our subsequent qualitative study (see below).

There may also be other reasons why we received a lower than expected number of referrals for the study. Some of the families may not have been referred because they did not speak sufficient English to provide consent. In Victoria, around 24% of families of children with congenital hearing loss are culturally and linguistically diverse; the exact proportion of families who do not speak sufficient English is not known (Z. Poulakis, Victorian Infant Hearing Screening Program, personal communication, 29th Nov 2021). It is also possible some children with complex medical needs were not referred. In an audit of a Victorian clinical service for children with hearing loss, nine out of 129 (7%) of children with mild hearing loss had complex medical needs (29). As this was a clinical service for children with medical needs, we would expect this proportion to be lower at a population level.

Our use of threshold averages for determining hearing loss degree was consistent with other studies that included children with mild losses (30). The design of our trial where we excluded children who were already fitted or had already decided about fitting hearing devices meant that most referrals for recruitment were for young infants, where it was necessary to rely on objective evoked potential threshold estimates. Our strict exclusion of children who had hearing thresholds outside the desired range at any frequencies aimed to maximise the rigor of the RCT. However, a more pragmatic approach to accommodate potential uncertainties around diagnostic thresholds, particularly for evoked potential threshold estimates, may have allowed for more referrals for consideration for recruitment in a real-life setting. Since the completion of our RCT, others have demonstrated the utility of unaided audibility to identify those children who, without amplification, may be at risk of language delays (31).

Two families participated in the RCT. Both families completed baseline and follow up data collection. The family randomised to the intervention group completed five out of six of the compliance questionnaires. Even though there was only one participant in the intervention group, monitoring device use by a short monthly parent report in REDCap may be an acceptable compliance monitoring method. The two participant families indicated that the experience of taking part was either positive or neutral, and completed the study protocols without issue. Although it was not possible to draw conclusions from two participants, there was no indication that the families found participating difficult or onerous. The low number of participants meant we could not address our primary aims.

To further understand the barriers and enablers to participation in an RCT, we subsequently conducted a qualitative study to understand perceptions of participation, by interviewing parents of children <2 years old with newly diagnosed bilateral mild hearing loss, and audiologists. We demonstrated the overarching facilitator to participation of parental and audiologists' desire to contribute to research to help determine whether hearing devices should be offered to newborns with mild hearing loss. This was in the setting of most parents and audiologists experiencing the stresses and challenges of uncertainty in the early management of mild hearing loss, congruent to previous research (18, 23). Individual circumstances, including how the family was coping with the new diagnosis, and their hearing aid preferences, could strongly influence their preferences for participation. Past studies on paediatric cancer trials have indicated that when consent for a child's participation in a trial is sought from parents soon after diagnosis, parents are likely to make decisions when they are distressed and vulnerable (32).

The strongest barrier, and perhaps the least modifiable factor, to participating in an RCT of hearing device fitting, was parents' reluctance for their child to be randomised to a treatment group, due to parental perception of losing control over choice of hearing device fitting. This was also against audiologists' values of family-centred practice. The perception of guilt of potentially causing harm to their child by not fitting hearing devices early was a notable barrier to participation. This has been shown in previous research, where anticipation of possible regret often accompanies a parent's sense of responsibility to protect their child in their decision-making while considering participating in trials (33). These may be challenges that cannot be easily overcome. In addition, there may be potential ethical concerns with not providing amplification for these children when failure to do so could potentially negatively impact communication development.

Nevertheless, we have learnt there are some potentially modifiable factors to improve uptake in research studies involving families of infants and young children with mild hearing loss. Parental perceptions of their child being a “guinea pig” in research could be addressed by increasing general awareness of research and better or clearer information about the study methodology and what was involved. Involving parents as study recruiters could also be a way to breakdown misconceptions and improve uptake rate. Potentially modifiable factors to study design to improve study uptake could include relaxation of eligibility criteria and increasing awareness amongst other child hearing health stakeholders (e.g., early intervention services, maternal child health nurses) of the study. The benefits of utilising clinicians as recruiters are many; however, it is important that researchers consider the burden they may be placing on busy professionals. In particular, we note that no ENT specialist or paediatrician made a referral for study recruitment. Many RCTs rely on clinicians as recruiters, with up to 50% failing to recruit target numbers (34). A 2013 systematic review identified 11 qualitative studies that centre around 8 themes relating to clinician's involvement and recruiting to RCTs; these would be of salience for any future trials in this population (34). A strong relationship and open communication between clinicians and the research team, and remunerating clinicians as recruiters (including protected time added to appointments to discuss the study) are paramount. Identifying clinicians with a passion for the study (such as participant A403), and further supporting their role in recruitment may also be a strategy that could improve parental uptake of the trial. It is possible that stronger engagement with diagnostic audiologists may have improved referral rates. In the state of VIC, we bypassed the need for diagnostic audiologists to refer to the study team by identifying potentially eligible participants through the state's newborn hearing screening program; extending this method to the other states may have helped. Nevertheless, even if referrals to the study were increased, the 82% parental decline rate means that unless we could address the reasons for parental decline, we would unlikely have been successful with recruiting enough participants.

Our study's greatest limitation was the inability to recruit sufficient participants for the RCT. Its strength was to use qualitative methodology to identify factors influencing participation in a RCT on hearing device fitting in infants with mild bilateral hearing loss, and in so, engaging the community in future study design. This research question is not answerable through a RCT design as the removal of parental choice through randomisation may not align with family centred practice. Therefore, alternative methodologies must be considered. These may involve novel methods of measuring infants’ ability to hear [e.g., objective assessments which measure speech discrimination in infants (35, 36)], and analysing outcomes data of aided and unaided infants with mild hearing loss from large observational studies, such as the proposed National Health and Medical Research Council (NHMRC) funded Australian National Child Hearing Health Outcomes Registry (ANCHOR), which aims to link data from child hearing health services in Australia to track child hearing outcomes.^1^

In conclusion, our attempted trial highlighted many barriers and challenges around trial recruitment involving randomisation for families at a very vulnerable and stressful time of their children's lives, shortly after their hearing loss diagnosis. Community engagement is paramount in designing and conducting research to determine whether early amplification benefits infants and young children with bilateral mild hearing loss. Important lessons have been learnt from the failure of recruitment for our proof-of-concept RCT. Better engagement of audiologists as recruiters, and additional supports for parents, may be necessary to improve recruitment rate in designing future studies. However, the RCT methodology takes away caregiver choice and control, may not align with family centred practice and may present a potential ethical concern for future adverse consequences if early amplification is not offered. Alternative research methodological approaches without randomisation are ultimately required to answer the important question of whether early hearing amplification benefits infants with mild bilateral hearing loss.

## Data Availability

The datasets presented in this article are not readily available because Data collected in this study is subject to the National Acousitc Laboratory's Privacy Policy, which can be found here: http://www.hearing.com.au/privacy-policy/. Participant information collected remains strictly confidential. Only the researchers directly involved with the study can access the information. We have undertaken to disclose the information only with expressed written permission from the participants, except as required by law. Requests to access the datasets should be directed to valerie.sung@rch.org.au.

## Footnotes

^1^ https://www.mcri.edu.au/research/projects/anchor
