# Supplementary material for: Mild matters: trial learnings and importance of community engagement in research for early identified bilateral mild hearing loss
Source: Front Pediatr. 2023 Aug 8;11:1197739. doi: 10.3389/fped.2023.1197739 (PMC10442842; doi:10.3389/fped.2023.1197739)
Supplement: Supplementary file 1 [file Datasheet1.pdf]

Supplementary Material

Mild Matters: trial learnings and importance of community engagement in research for early identified bilateral mild hearing loss

Valerie Sung*, Teresa Y.C. Ching, Libby Smith, Vivienne Marnane, Michelle Saetre-Turner, Alison King, Rachael Beswick, Claire E. Iseli, Peter Carew

*** Correspondence:** Valerie Sung: valerie.sung@rch.org.au

Supplementary Interview Guides

Caregiver semi-structured interview guide:

As we discussed before, we found that many parents find it hard to decide whether their baby with mild hearing loss should have hearing aids or not. This is mainly because we don't know whether the hearing aids are helpful when the babies are so young. Last year, we tried to answer this question by doing a research study. We offered parents like you the chance to participate in a research study where their baby is either fitted with hearing aids, or not fitted, for a period of 6 months. After this time, the parent could choose whether to continue with hearing aids or no hearing aids for their baby. We randomly allocated whether their baby was fitted with hearing aids or not – that is, there was a 50% chance that their baby would have hearing aids fitted, and 50% chance their baby would not have hearing aids fitted.

We are interested to know how you would feel if you were, hypothetically, offered this choice. So what we are about to discuss is hypothetical – we will not actually be asking you to take part in any other research project today. Does this all make sense so far?....(No – answer more questions; Yes – continue)

a. First of all; what kind of information would help you decide on whether you would participate in such a research study (where you would 'flip a coin' and your baby would either be fitted with hearing aids or not)?
b. If you were asked to take part in the study where your child would be randomly allocated to wear hearing aids or not wear hearing aids for 6 months, would you agree to take part? Yes/no, why/why not
c. What reasons can you think of that would make parents want to take part in a study like that?
d. What reasons can you think of that might stop other parents from wanting to take part in a study like that?
e. If we knew that wearing hearing aids was safe, but didn't know if it was effective, would you change your mind?

That is the end of the interview. Is there anything else you would like to share before we finish?

Thank you very much for taking part!

Audiologist semi-structured interview guide:

When you see a baby with a new diagnosis of bilateral mild hearing loss, what are your thought processes and what is your approach to counselling and managing the family?

1. How would you manage the baby?
2. What factors help you decide on your management?
3. Can you tell me about the last time you saw a baby with a mild bilateral hearing loss. What decisions were made about treatment or what did you talk about with the family?
4. What do you think is the evidence in managing infants with mild bilateral loss?

I would like you to think of a research study where babies diagnosed with mild bilateral hearing loss are randomly assigned to have a hearing aid fitted or not, for a period of 6 months.

1. What information do you think a study like this may provide?
2. Do you think a study like this may be useful? Why/why not?
3. If you were asked to provide eligible families with information about a study like this, how would you feel?
   a. How comfortable would you be to provide the information?
   b. What may prevent you from giving families information about a study like this?
   c. What would help you provide families with information about a study like this?
   d. How would you feel if you disagreed with the treatment the child was randomized to receive? What would you do?

Supplementary Table 1. Trial referral sources and state of residence.

State	VIHSP [1]	Hearing Australia Audiologist	Diagnostic Audiologist	Total
VIC	13	-	2	**15**
NSW	-	16	-	**16**
QLD	-	7	2	**9**
**Total**	**13**	**23**	**4**	**40**

[1]Victorian Infant Hearing Screening Program.

Supplementary Table 2: Baseline characteristics of trial participants

	Intervention, n=1	Control, n=1
Age at enrolment (months)	6.2	3.3
Sex	Male	Female
Age at diagnosis (months)	3.7	0.6
Gestation (weeks)	41	40
SEIFA disadvantage index [1]	1020	964

Maternal education	Completed Yr 12	Completed Yr 12
Primary Language	English	English

[1]SEIFA = Socio-Economic Indexes for Areas Index of Relative Socio- Economic Disadvantage. It is a composite Census-based measure summarising the social and economic conditions of Australian neighbourhoods (national mean 1000, SD 100, where higher values represent less disadvantage). https://www.abs.gov.au/websitedbs/censushome.nsf/home/seifa

Supplementary Table 3. Trial participant outcomes (total n=2)

**Domain**	**Measure**	**Intervention n=1**	**Control n=1**
**Primary outcome: Child**			
Language	CSBS Scale: Speech composite scaled score (percentile rank)	5 (5th)	12 (75th)
**Secondary outcomes: Child**			
Social skills	CSBS social composite scaled score (percentile rank)	8 (25th)	7 (16th)
	CSBS symbolic composite scaled score (percentile rank)	5 (5th)	7 (16th)
Functional Performance	PEACH-plus Quiet Score	50	70
	PEACH-plus Noise Score	40	60
	PEACH-plus Overall Score	45	65
Listening Effort	PEACH-plus Quiet Score	45	70
	PEACH-plus Noise Score	45	65
	PEACH-plus Overall Score	45	68
**Secondary outcomes: Parent**			
Psychological well-being	Parenting Morale Index raw score	40	43
Parent-child relationship	FACDD raw score	51	45
Quality of Life	AQoL-4D standard score	86	89
